# Overview of naturally occurring radioactive materials (NORM) in mineral processing industries and consideration of ionising radiation in life cycle assessment – Part I. NORM inventory

**DOI:** 10.1007/s00411-025-01144-0

**Published:** 2025-09-17

**Authors:** Alla Dvorzhak, Raffaella Ugolini, Gennaro Venoso, Federica Leonardi, Boguslaw Michalik, Almudena Real, Alicia Escribano, Danyl Pérez-Sánchez, Nathalie Vanhoudt, Antti Kallio, Cristina Nuccetteli, Flavio Trotti, Rosabianca Trevisi, Laureline Fevrier, Jelena Mrdakovic Popic

**Affiliations:** 1 Research Centre on Energy, Environment and Technology (CIEMAT), Av. Complutense 40, Madrid, 28040 Spain; 2 Environmental Protection Agency of Veneto (ARPAV), Verona, Italy; 3 National Institute of Health (ISS), National Centre for Radiation Protection and Computational Physics, Rome, Italy; 4 National Institute for Insurance Against Accidents at Work (INAIL), DiMEILA, Monteporzio Catone, Rome, Italy; 5 Central Mining Institute - National Research Institute, Silesian Centre for Environmental Radioactivity (GIG- PIB), Plac Gwarków, 1, Katowice, 40-166 Poland; 6 Belgian Nuclear Research Centre (SCK CEN), Biosphere Impact Studies, Boeretang 200, Mol, 2400 Belgium; 7 Radiation and Nuclear Safety Authority (STUK), Valtakatu 15-17, Rovaniemi, FI-96200 Finland; 8 Autorité de Sûreté Nucléaire et de Radioprotection (ASNR), PSE-ENV/SPDR/LT2S, Saint-Paul-lez- Durance, F-13115 France; 9 Norwegian Radiation and Nuclear Safety Authority (DSA), Grini Naeringspark 13, Østerås, Norway

**Keywords:** Natural radionuclides, RadoNorm project, Radioecology, Radiological impact assessment, NORM-involving mineral industries

## Abstract

A systematic overview of NORM related issues in mineral processing industries as production of phosphoric acid, titanium dioxide (TiO_2_) pigment, phosphate fertilizers, cement and zircon and zirconium industry in different European countries is provided in this work. The current study tracks composition, content of Naturally Occurring Radionuclides (NOR) and their changes throughout the entire production cycle in these common and important industries: from raw materials, different production processes, end products, residues and wastes, and releases, with analysis of possible subsequent use of some by-products and residues in the concept of the circular economy.Such work improves the radiation protection knowledge in mineral processing industries, assists in the management of generated residues and wastes, and supports the regulatory decision-making process. A significant novelty is ensured through comprehensive comparison of the given mineral industries and respective NORM issues in various aspects – from legislative frameworks applied, NORM matrices with different NOR activity concentration and possible challenges with respect to recovery or disposal when they are above exemption or clearance levels of 1 kBq kg^−1^. This type of a systematic and comprehensive overview is especially important for European countries regarding the framework of Life Cycle Assessment (LCA) and the circular economy, where resources are maximally used, the involvement of new ones is reduced, waste is avoided, and the life cycle of products is extended. Additionally, here presented inventory work is expected to be useful for many countries worldwide that are at the beginning of their NORM-involving practice mapping.

## Introduction

Radiation protection issues related to the Natural Occurring Radioactive Material (NORM) have been studied for decades (Gesell 1975; Testa et al. 1994; Kathren 1998; Underhill 1996; IAEA 2022, 2024; Mrdakovic Popic et al. 2025). Eventually, recent EU regulation of Council Directive 2013/59/Euratom (European Commission 2014) explicitly requires Member States to include regulations on exposure to naturally occurring radionuclides (NOR) in their national legislation. This highlights the importance of conducting additional research on NORM with regards to different industrial practices but also various NORM related aspects (Mrdakovic Popic et al. 2025). The RadoNorm research project was initiated in 2020 within the framework of the Euratom research and education programme to address existing knowledge gaps in hazard management related to radon (Rn) and NORM exposure situations. The aim is to ensure effective radiation protection (RP) through improved scientific evidence and social considerations (Kulka et al. 2022). One of the project’s research outputs is the development of a methodology and tools for the exhaustive inventory and characterization of NORM involved situations (Michalik et al. 2023; Mrdakovic Popic et al. 2023a). This methodology is based on four tiers, enabling a systematic review of potential situations causing significant exposure to natural radiation, starting with ore bodies’ characterization, and ending with end-product use and waste treatment. The third tier of the methodology focuses on mineral processing industries following extractive industry.

In mineral processing, valuable minerals are separated from ore and concentrated for future use. Applied processes are based on many technologies including either physical or chemical fractionation, inorganic chemistry, pyrometallurgical processes (calcining, roasting, refining), hydrometallurgy (leaching, solution concentration and purification), metals smelting and fossil fuels combustion but are not limited to. NOR from raw materials can accumulate during these processes, requiring investigation to identify materials classified as NORM. As radioactivity is not the property for which these materials are processed for, accumulation of NOR is mainly observed in solid residues, liquid and/or gaseous effluents discharged into the environment. However, there are also some situations where NOR in relatively high concentrations may be present in final products (e.g. fertilizers, refractories, or abrasives). Such enhanced activity concentration of NOR is a primordial property of the minerals, which these products were originally made out of. Notwithstanding this, identified NORM should be fully characterized, considering their role, total abundance, chemical and physical properties, and NOR activity concentration to address radiation protection concerns. Potential harmful effects on human health and the environment due to NORM should be identified and then investigated for radiation protection purposes. Such knowledge is important also in the context of Life Cycle Assessment (LCA) and the circular economy (European Commission 2020). LCA, in accordance with EU consortium for life cycle impact assessment of a product or service over the course of its entire life, should consider various impact categories, with regard to protection of climate, environmental and human health, however impact of NORM is rarely considered to date.

In the context of the RadoNorm project, mineral processing industries such as phosphoric acid production, titanium dioxide (TiO_2_) pigment production, phosphate fertilizers, zirconium industry, and cement industry from different European countries were analysed. Industrial sectors of this kind typically utilise feedstock or raw materials with relatively elevated levels of NOR. Consequently, regardless of the subsequent processes employed, these materials are classified as NORM. Furthermore, the principal processes responsible for NOR fractionation and accumulation differ somewhat from those used in either energy production from fossil fuels or in extractive metallurgy, particularly pyrometallurgy which are intended to be addressed in dedicated articles.

The main objectives of this study are to:

- Present new and updated NORM data (inventory), including the main features of the processes and existing hazard evaluation based on afore mentioned industries, which were previously mapped as most frequent NORM involving industries across European countries (Mrdakovic Popic et al. 2023b) and propose potential use of this NORM inventory in terms of a LCA for the products of mineral processing industries (procedure will be presented in detail in the paper II, part of this overall work);
- Provide a useful overview of NORM matrices where some NOR are present at levels above the legislative requirements of exemption level, and identify critical points and future challenges.

In alignment with the objectives of this study, the authors highlight that the findings will provide substantial support to countries at varying levels of regulatory control for NORM.

## Materials and methods

The data analysed in this study were collected during the period 2022–2024 using: (a) the developed identification methodology for NORM exposure situations (Michalik et al. 2023), (b) data collection tools (Mrdakovic Popic et al. 2023a), (c) a NORM survey conducted in European countries (Mrdakovic Popic et al. 2023b).

**Fig. 1:**
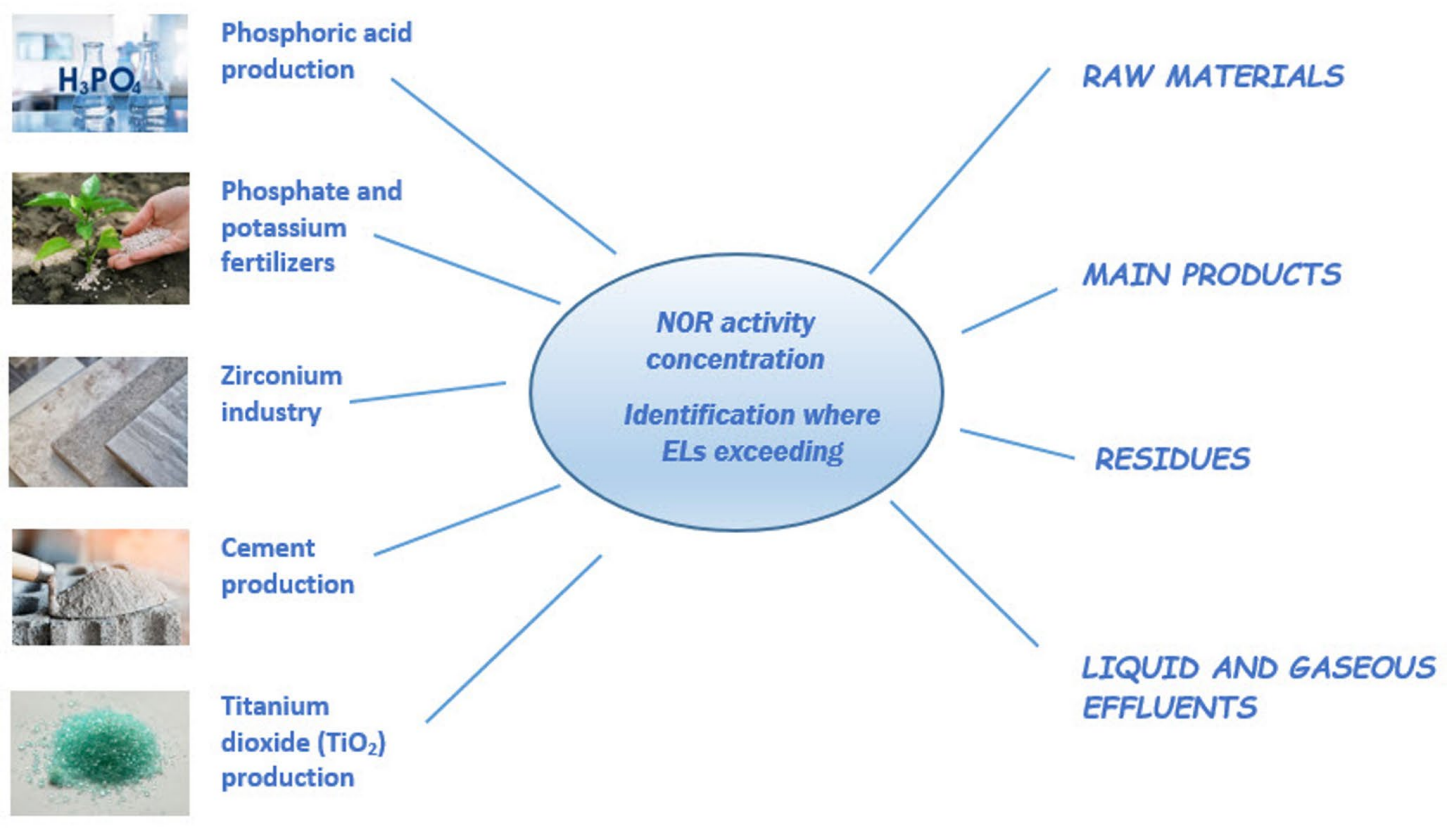
Analysis of NOR activity concentration in mineral processing industries Activity concentration of NOR in different materials are provided in tables of Sect. 4 as ranges or mean values. Since data from RadoNorm participating countries were collected differently (as maximum, minimum and mean values), the authors decided to present the pooled data either as ranges (where possible) or as mean values

Data for mineral processing industries (Fig. 1), subject to evaluation in the current work, were collected, from Belgium, Finland, France, Italy, Norway, Poland and Spain, which participated in the RadoNorm project. In addition, these data were compared with data from other European countries obtained by the e-NORM survey (Mrdakovic Popic et al. 2023b). More detailed information on data collection conditions are given elsewhere (Mrdakovic Popic 2023a, b; Mrdakovic Popic et al. 2025).

## Legislative and regulatory aspects for NORM

First legislation in natural ionising radiation was introduced in Europe in 1990 s, which triggered more interest in research and regulation. The EU Directive 2013/59/Euratom, EU BSS, explicitly introduced requirements for natural radiation to be considered and regulated like ionising radiation from artificial radionuclides (European Commission 2014). It specifically included NORM-involving industries, exposure to radon and the control of NOR in building materials. European member states, must ensure the identification of classes or types of practice involving NORM and leading to exposure of workers or members of the public, which cannot be disregarded from a radiation protection point of view, develop graded regulatory control approach to ensure radiation protection by, among other measures, adopting and implementing into national regulations the numerical values of exemption and clearance levels (EL and CL, respectively) for solid NORM expressed as activity concentration (1 kBq kg^−1^ for ^238^U and ^232^Th decay chains, and 10 kBq kg^−1^ for ^40^K), as well as a dose criterion of 1 mSv y^−1^ from all NOR considered as a justified and legally accepted increment of effective dose above natural background. An indicative list of NORM-involving industries was also published to help Member States initiate the necessary actions.

The information collected in the NORM survey conducted in EU countries (Mrdakovic Popic et al. 2023b) showed that EU Member States have developed their own, often country specific approaches for the NORM-involving industries investigated here, some even before the EU BSS of 2014. These approaches include various elements – for instance application of specifically developed set of CLs, use of a summation rule, use of dose limit criterion below 1 mSv y^−1^, different types of control and monitoring programmes for discharged liquids and gases. That is considered as reflection of a certain flexibility given in the EU BSS, since countries may and do have different industries that are regulated in different ways, as well as different infrastructures and disposal facilities. This means that they also have different approaches to waste treatment. More details on legislative frameworks implemented and examples on developed approaches can be found elsewhere (The Scottish Government 2014; The Radiation Safety Authorities, 2023; Van Velzen and Welbergen 2016; RP 122 2001; Gellermann et al. 2023).

The issue of NOR in waste is particularly differently considered in different countries. In some countries, all waste containing NOR above 1 kBq kg^−1^ are to be considered as radioactive waste and must be properly disposed with specific requirements within RP regulatory control, although not necessarily in radioactive waste repositories. In many other countries, NORM have been considered to have much more similarities with the common industrial waste than with radioactive ones (Michalik 2009; Mrdakovic Popic et al. 2023b). The interdependence of existing legal provisions regulating the management of industrial waste and radioactive waste in the context of their use to determine the procedure for handling NORM waste is discussed in detail in the RadoNorm project deliverable 5.13 (Michalik et al. 2025).

## Main features of NORM in mineral processing industries

### Phosphoric acid production

Phosphoric acid is produced from phosphate rock, and its main application is as a raw material for fertilizer manufacturing (CSN 2011; Bolívar et al. 2009a). The most common type of wet process used for phosphoric acid production is based on the reaction of phosphate rock with sulphuric acid. Phosphate rock contains variable amounts of NOR and when it is digested with acid, some of them, particularly radium isotopes, concentrate in residues, such as the scale that tends to form inside pipes and vessels (Bolívar et al. 2009b; CSN 2011; Guerrero-Márquez, 2017). In high-temperature processes, radioactive isotopes of the more volatile elements (polonium and, to a lesser extent, lead), become concentrated in the fume. Hence, from a radiation protection point of view, process residues potentially pose a greater concern than the original raw materials and products (IAEA 2013).

The production of phosphoric acid through the wet process generates a solid waste called phosphogypsum (PG), in which ^226^Ra and ^228^Ra can reach up to 0.7 and 0.3 kBq/kg respectively, and ^210^Pb – up to 1.2 kBq/kg (Saueia et al. 2005). About 5 tons of PG are generated per ton of phosphoric acid produced, and worldwide PG generation is estimated to be around 100–280 Mt per year. PG is mostly disposed of without any treatment, usually by dumping it in large stockpiles (Tayibi et al. 2009) that occupies large land areas and causes serious environmental problems including radioactive contamination. Its storage at open piles, exposed to various weathering conditions, may lead to radioactive contamination of the environmental system, what has been shown in different studies worldwide (Alcordo and Rechcigl 1993; Rutherford et al. 1994; Attar et al. 2011).

PG primarily occurs in the form of CaSO_4_·2H_2_O, and besides NOR also contains impurities of environmental concern, such as residual acids, fluoride, and heavy metals. The composition of these impurities can vary greatly depending on the origin of the phosphate rock used in phosphoric acid production (Akfas et al. 2024). In line with circular economy principles, waste such as PG should be recovered as raw materials, for example in construction materials or other applications. However, approximately 58% of PG is stacked, 28% is discharged into coastal waters, and only 14% is further processed (Bilal et al. 2023). The recovery of PG may increase in the future, and a systematic study and control of its entire production are necessary, particularly regarding to its environmental and health impacts.

Even though PG is the most critical waste in the phosphate fertilizer industry due to large volumes (detailed analysis done in the next section), other NORM, in the form of scale, sludge, and discharge into air and water bodies, should not be neglected, especially given the high activity concentration of certain NOR and the potential risks when handling and disposing of them, or once they enter the environment.

Among investigated radionuclides, ^238^U, ^232^Th and their progeny,^226^Ra was found to dominantly accumulate in other residue types, mostly in scales and sludges, significantly exceeding the legislative values from the EU BSS, which consequently made them not only NORM waste, but radioactive waste in many European countries (HERCA 2021). These materials, in comparison to PGs were present in significantly lesser volumes, but as they pose an occupational hazard, safety and radiation protection measures must be applied when handling them. Such practice was reported in all countries participating in this study. Scale and sludge material with enhanced NOR activity concentration are disposed at dedicated disposal sites with special radiation protection requirements.

The data collected in this study regarding to raw materials, end-products, and residues from the phosphoric acid production industry are presented in Table 1.

**Table 1:** Activity concentration of relevant radionuclides in different fractions of radiological concern related to phosphoric acid production

Phosphoric acid production								
Material type	^238^U	^226^Ra	^210^Pb	^210^Po	^232^Th	^228^Ra	^228^Th	^40^K
*1. Raw materials [kBq kg*^*−1*^]								
Sedimentary rock (Morocco)	**1.2**–**1.7**	**1.2**–**1.7**	**1.5**–**1.7**	–	0.02–0.04	0.02–0.022	0.017–0.023	–
Igneous rock (Kola)	0.07–0.08	0.065–0.07	0.04–0.06	–	< 0.04–0.1	0.1–0.11	0.09–0.1	–
Potassium salt	< 0.1	< 0.007	–	–	< 0.1	< 0.003	< 0.001	**14.4**
*2. Main products [kBq kg*^*−1*^]								
Phosphoric acid (H_3_PO_4_)	0.01–0.7	–	–	–	–	–	–	–
Phosphoric acid 54% P_2_O_5_ (merchant grade)	**1.7**–**2.0**	0.007–0.008	0.1–0.12	–	–	< 0.001	0.01–0.02	0.01–0.02
*3. Residues [kBq kg*^*−1*^]								
Phosphogypsum	0.01–0.3	0.003–**1.1**	0.002-0.7	–	0.02–0.05	0.004–0.02	0.002	–
Scales	0.01–**1.0**	0.002–**1.4**	**2.0**	–	–	0.002	0.001	0.018
Gypsum	0.3–0.6	0.6–0.7	0.6–0.64	–	0.02–0.03	0.009–0.01	0.007–0.01	–
Liquid Sludge	**1.1**–**2.3**	0.004–0.008	0.04–0.09	–	0.002–0.03	0.002–0.002	0.004–0.02	–
Solid Sludge	0.8–**1.3**	0.7–**4.9**	**1.3**–**2.0**	–	0.009–0.03	0.008–0.07	0.006–0.04	–
Filters	0.6	**2.1**	**1.0**	–	–	–	–	–
*4. Liquid and gaseous effluents [kBq kg*^*−1*^]								
Waters	0.2–0.22	0.003–0.004	0.07–0.12	–	0.0004–0.0007	< 0.002	0.001–0.002	–

Activity concentrations higher than EU BSS ELs & CLsare highlighted in bold

Raw materials, main products and residues with activity concentrations higher than 1 kBq kg^–1^ for ^238^U and ^232^Th series, and 10 kBq kg^–1^ for ^40^K (for at least one radionuclide) are highlighted in bold

Regarding the **raw materials**, activity concentrations higher than 1 kBq kg^−1^ for at least one radionuclide have been found for sedimentary rock from Morocco, while regarding the **main products**, activity concentrations higher than 1 kBq kg^−1^ have been found for 54% phosphoric acid for ^238^U.

Regarding the **residues** according to the data collected in this study, the most significant NOR in PG waste is ^226^Ra, whose activity concentration vary widely and can reach up to 1.1 kBq kg^−1^. The activity concentration values presented in the Table 1 are comparable to published values (UNSCEAR 2000; IAEA 2013). Compared to results of the e-NORM survey, the upper level is in good accordance with the values presented by Mrdakovic Popic et al. 2023b while the low level can be significantly lower.

In terms of volume, the residue with the highest abundance was PG, as expected, still in a good accordance with values presented by other authors worldwide (Mazzilli et al. 2000; Abril et al. 2009; Attar et al. 2011). Regardless relatively low activity concentration of NOR, when exposed freely in the changeable environmental conditions, PG can pose a threat to the environment, due to the huge amount of material stored, as previously noted (Tayibi et al. 2009).

Data on the activity concentration of ^210^Po in Table 1 are unavailable. However, sources such as García-Tenorio (2012) suggest that its activity concentration in PG can range from 0.725 to 1 kBq kg^−1^. It is reasonable to assume that for two-year-old PG, the activity concentration of ^210^Po is equivalent to that of ^210^Pb, except in cases where there has been significant exchange of material with surrounding environmental compartments. It is important to track also the activity concentration in scale, as well as in liquid and solid sludges. NOR such as ^238^U, ^226^Ra and ^210^Pb are of highest importance in scale, where their concentrations can reach EL values or higher. For dry sludges, ^238^U, ^226^Ra and ^210^Pb are the most significant, while for sludge leakage, ^238^U is the main radionuclide of concern.

### Phosphate and potassium fertilizers industry

Current agriculture practices rely on the use of mineral-based (phosphate and potassium) fertilizers (Roberts and Johnston 2015). Commercial phosphate fertilizers are manufactured using both phosphate rock directly and phosphoric acid with certain inorganic salts and oxides as intermediate products from other processes. However, while rock phosphate has been a source of phosphorous for soils over long periods of time in the past, nowadays there are challenges such as the depletion of this source, limited phosphorous in some raw materials, high transportation costs, and low crop responses. On the other hand, the use of phosphate fertilizers has grown significantly worldwide (Mogollon et al. 2018; Zou et al. 2022). Similarly, commercial potassium fertilizers are primarily manufactured from potash minerals from underground rock deposits (e.g., carnallite, sylvinite, langbeinite, hartsalz). Although global potassium sources are stable, there are still existing technical and economic challenges when considering potassium fertilizers production. Most specifically, challenges are related to decrease of potash availability as deposits are concentrated in a few countries, what affects transportation, high energy and processing costs and products prices, and cause certain environmental concerns. All these facts contributed to the erratic production of potash with volatile prices (Mikkelsen and Roberts 2021).

Depending on the source of raw materials and manufacturing processes, various types of fertilizers are produced. The major manufacturing processes for both types of fertilizers are the same and include thermal route, wet chemical route, mining and physical route (flotation mainly for potassium chloride; minimal for phosphate fertilizers), precipitation and mixing (for complex chemical compounds fertilizers of both types) (Da Silva and Kulay 2005; Finch et al. 2014). The most common fertilizers are single superphosphate (SSP), triple superphosphate (TSP), fused magnesium phosphate (FMP), ammonium phosphates or mono- and di-ammonium phosphate (MAP, DAP), superphosphoric acid (SPA), potassium chloride (MOP), potassium sulphate (SOP), potassium nitrate and langbeinite and multi nutrient fertilizers (N-P-K) (Finch et al. 2014; Saueia and Mazzilli 2006).

Raw materials may contain different activity concentration of NOR, which is further reflected in a range of NOR activity concentrations in the various types of fertilizers and residues (Table 2).

**Table 2:** Activity concentration of relevant radionuclides in different fractions of radiological concern related to fertilizers industry

Phosphate fertilizers industry								
Material type	^238^U	^226^Ra	^210^Pb	^210^Po	^232^Th	^228^Ra	^228^Th	^40^K
*1. Raw materials [kBq kg*^*−1*^]								
Sedimentary rock (Tunisia)	**1.4**	0.8	–	–	0.02	–	–	0.04
Sedimentary rock (Morocco)	**1.0**–**1.5**	0.6–**1.5**	–	–	0.01–0.9	–	–	–
Phosphate ore	0.5–**1.2**	0.5–**1.4**	0.4–1.3	–	–	–	–	0.4
Potash ore	**1.0**	**1.0**	**1.0**	–	–	–	–	**13.4**–**15.1**
Apatite	0.01–**1.9**	0.012	–	–	0.021	–	–	0.029
Phosphoric acid	**1.65**–**2.0**	0.007–0.01	0.095–0.12	–	–	0	0.01–0.015	0.012–0.4
CaCO_3_	0.03	0.025	0.017	–	–	0	0.003	0.05
CaO	0.03	0.032	0.003	–	–	0.01	0.006	0.053
MgO	0.007	0.002	0.002	–	–	0	0.001	0.008
*2. Main products [kBq kg*^*−1*^]								
Monoammonium phosphate	**1.6**–**2.0**	0.046–0.05	0.044–0.076	–	–	0	–	0.01–0.016
Diammonium phosphate	**1.5**–**1.8**	0.04–0.05	0.217–0.277	–	–	0	–	0.01–0.017
NPK 15/15/15	0.5–0.6	0.007–0.009	0.23	–	–	0	–	3.8–4.2
NPK 8/24/8	0.8–0.9	0.03–0.034	0.23	–	–	0.007–0.01	–	2.1–2.3
Potash	0.01–0.02	0.001	0.17	–	–	0	–	**14.9**–**16.5**
Muriate of potash (MOP) (KCl) − 4 types of different origin	0.1	0.01	0.006	–	0.01	0	0.003	**13.8**–**16.0**
Urea	0.01–0.02	0.001	–	–	–	0	–	0.011
Potassium sulphate (K_2_SO_4_)	0.1–1.5	0.01–0.5	–	–	0.01–0.23	–	–	0.1–7.5
*3. Residues [kBq kg*^*−1*^]								
Phosphogypsum	0.06	0.001–0.45	0.01	–	–	0.01	0.006	0.009
Scale	0.03	0.003–**90.0**	0.003	–	–	0.01	0.02	0.492
Calcium fluoride sludge	–	**2.0**–**10.0**	–	–	–	–	–	–
Acid insoluble minerals – solid residues	0.01–0.02	–	**1.6**–**1.8**	–	–	–	–	–
*4. Liquid and gaseous effluents [kBq kg*^*−1*^]								
Discharge to air and sea	0.02–0.06	0.06–0.25	0.05–0.9	–	–	–	–	0.06−0.02

Activity concentrations higher than EU BSS ELs & CLsare highlighted in bold

Raw materials, main products and residues with activity concentrations higher than 1 kBq kg^–1^ for ^238^U and ^232^Th series, and 10 kBq kg^–1^ for ^40^K (for at least one radionuclide) are highlighted in bold

Results from the current study showed that raw materials used have elevated ^238^U and ^226^Ra concentration levels, higher than the ELs given in the EU BSS. Some phosphate ores also exhibit increased levels of ^210^Po, 1-1.3 kBq kg^−1^, which are comparable with previous values reported by other authors (Attar et al. 2011; Sahu et al. 2014). In contrast to phosphorus ores and phosphoric acid, other compounds (CaCO_3_, Cao, MgO), used in the fertilizers production, do not have elevated NOR concentrations, i.e. they are lower than the ELs and CLs of the EU BSS (Table 2). Potash ores are rich in potassium and have high ^40^K levels, exceeding 10 kBq kg^−1^ (Kim and Cho 2022). In the EU, only Germany is exploiting potassium rich mineral in noticeable amount, and significant part is exploited from underground seams by shaft-mine technology. Mine output, which contains some proportion of potassium minerals, at least more than 20–25%, is subject to beneficiation locally. Finally, potassium concentrates available on market are almost pure KCL, hence there is no other radionuclides than ^40^K. On the other hand, there are neither data about NOR in waste from comminution process nor occupational exposure of miners involved in potash mining.

Activity concentration of NOR in the main products – fertilizers were found to be low in majority of products, with exception of mono- and diammonium phosphate and potassium sulphate that had slightly elevated ^238^U levels, up to 2 kBq kg^−1^, above EU BSS numerical values for ELs and CLs.

In the context of fertilizers based on phosphate rocks, the target element is phosphorus. NOR are enclosed within the crystal lattice of phosphates due to the efficient diadochy process. This radioactivity should be regarded as a form of impurity. On contrary, in fertilizers based on potassium minerals, radioactivity is an inherent part of its composition, as potassium is the target element. Natural potassium is a mixture of potassium isotopes, of which only ^40^K is radioactive, accounting for 0.0117% of the total mass of potassium. This indicates that 1 kg of natural elemental potassium has ^40^K activity of approximately 31 kBq, and the activity of potassium compounds is contingent on the mass ratio of included nuclides. For instance, the activity concentration of KCl is estimated to be around 16.5 kBq/kg. Consequently, it is not feasible to produce potassium fertilizers that are free of radioactivity, which is at least theoretically possible in the case of phosphate fertilizers.

From this perspective, exposure to potassium fertilizers should be regarded as a part of natural background. Nevertheless, given that potassium minerals are incorporated into technological processes, related exposure should be evaluated in terms of radiation protection. It is worth noting that, regardless the process, there is no enhancement of activity concentration, and, consequently, MOP and potash are characterised by ^40^K levels up to 16.5 kBq kg^−1^.

### Zirconium industry

Zirconium occurs in nature as zirconium oxide or zirconia (ZrO_2_) in the mineral baddeleyite but most commonly as zircon sand, i.e. in the form of zircon or zirconium silicate (ZrO_2_⋅SiO_2_). Due to the scarcity of natural zirconia, most of the zirconia now available is manufactured from zircon, by subjecting zircon sands to specific thermal and chemical processes (IAEA 2007).

The uranium and thorium decay series radionuclides in zircon are normally regarded as being in equilibrium with their parent radionuclides, while in baddeleyite and zirconia there is a less likelihood of decay chain equilibrium (IAEA 2007).

Zircon and zirconia are widely used in various industrial sectors due to their important physical and chemical properties (IAEA 2007).

The material’s applications are diverse, encompassing the production of refractory materials, the use as ingredients in glazes for ceramic tiles and sanitary ware, and in the composition of certain stoneware types. Its utilisation extends to the chemical industry, the fabrication of abrasive materials, and precision foundry, where it is employed in the manufacture of various artefacts. In the metal casting industry, it is used as foundry sand and for the cleaning of moulds. It is also used in the manufacture of laboratory crucibles capable of withstanding high thermal shocks, for furnace linings in metallurgy, and in microelectronics and semiconductor components. Furthermore, it is used in bioceramics for bone prostheses and dental ceramics.

Data collected within RadoNorm project comes mainly from the industrial sector of refractory and ceramic production, which is the largest application of zircon and zirconia according to IAEA (2007).

Information was collected on the production of refractory bricks and materials for direct use in furnaces in the glass and steel industries, the production of objects by precision casting, the production of sanitary ware and stoneware.

Data regarding activity concentration of NOR in raw materials, products and residues related to the zirconium industry are presented in Table 3.

**Table 3:** Activity concentration of relevant radionuclides in different fractions of radiological concern related to zirconium industry (refractory industry and tile industry)

Zirconium industry (refractory industry and tile industry								
Material type	^238^U	^226^Ra	^210^Pb	^210^Po	^232^Th	^228^Ra	^228^Th	^40^K
*1. Raw materials [kBq kg*^*−1*^]								
Zircon sands (ZrSiO_4_, zirconium silicate)	**1.1**–**5.6**	**1.2**–**23.5**	< 0.3–**4.3**	**2.6**	0.4–**2.1**	–	–	< 0.1–0.1
Zirconium oxide (ZrO_2_)	**3.2**–**13.4**	**1.2**–**5.6**	0.3–**1.0**	–	0.4–**2.7**	–	–	< 0.1–0.3
Silica Fume	0.8	**4.4**–**6.4**	–	–	0.2–0.4	–	–	4.6–6.2
Bauxite	0.4	–	–	–	0.3	–	–	0.1
Calcined Bauxite	0.5	0.4	0.2	–	–	0.5	0.5	0.1
Chamotte	0.1–0.2	0.1	0.1	–	0.1–0.2	0.2	0.2	0.1–0.3
Silicium carbide		0.2	–	–	0.1	–	–	0.1
*2. Main products [kBq kg*^*−1*^]								
Refractory	0.2–**2.7**	–	–	–	< 0.1–0.6	–	–	0.1–0.4
Refractory slurry	**2.2**	–	–	–	0.7	–	–	0.1
Refractory concrete	0.2–0.5	–	–	–	0.3–0.5	–	–	0.1–0.2
Porcelain stoneware	< 0.1	–	–	–	< 0.1	–	–	0.5–0.7
White porcelain stoneware	0.2	–	–	–	< 0.1	–	–	0.5
*3. Residues [kBq kg*^*−1*^]								
Fusion furnace dust	0.4–0.9	0.2–0.3	**21**–**37**	**35**–**126**	< 0.1	–	–	< 0.1
Hydrated lime	< 0.1	< 0.1	< 0.1–0.4	**5**–**46**	< 0.1	–	–	< 0.1–0.4
General air exhauster dust	< 0.1–**1.2**	< 0.1–**1.3**	0.1–**3.0**	0.1	< 0.1–0.2	–	–	< 0.1–0.5
Scrap grinding dust	0.5–**1.2**	0.5–**1.1**	0.5–**1.0**	**1.3**	0.1–0.2	–	–	0.3
Refractory cooling dust	< 0.1	< 0.1	< 0.1	0.2	< 0.1	–	–	< 0.1
Sludge	< 0.1–**2.0**	0.1–**2.0**	0.1–**1.2**	0.1–**1.2**	< 0.1–0.4	–	–	< 0.1–0.4
Scrap refractory residues	0.9–**1.6**	**1.6**–**4.3**	0.8	–	0.2–**1.2**	–	–	< 0.1–0.2
Scrap ceramic residues	0.1	0.1	0.1–0.2	–	< 0.1	–	–	0.4–0.6
Enamel suction filters	0.2	–	–	–	< 0.1	–	–	0.4–0.5
*4. Liquid and gaseous effluents [kBq kg*^*−1*^]								
Mill recovery water	< 0.1	–	–	–	< 0.1	–	–	< 0.1

Activity concentrations higher than EU BSS ELs & CLs are highlighted in bold

Raw materials, main products and residues with activity concentrations higher than 1 kBq kg^–1^ for ^238^U and ^232^Th series, and 10 kBq kg^–1^ for ^40^K (for at least one radionuclide) are highlighted in bold

Regarding **raw materials**, activity concentrations higher than 1 kBq kg^−1^ for at least one radionuclide have been found for *zircon sand*s, *zirconium oxide* and *silica fume*. The data presented in the table is largely consistent with the data reported in a previous review (IAEA 2007). Notably, silica fume is produced in the reduction process of fusing zirconia from zircon sands. The high temperatures used in the process destroy the crystal structure and allow the more volatile elements such as ^226^Ra, ^210^Pb and ^210^Po to be partially removed in the silica fume. There is some evidence to support this, with high values of ^226^Ra reported in Table 3 and significant levels of ^210^Pb and ^210^Po (of about 10 kBq kg^–1^) reported in literature (IAEA 2007). Instead, bauxite, calcinated bauxite, chamotte and silicium carbide materials seems to be not radiologically relevant confirming results of other studies (UNSCEAR 2000; IAEA 2007; Righi 2009).

Regarding the **main products**, it is worth noting that their activity concentrations are related to the type, quantity and origin of raw materials. For refractories, elevated levels of ^238^U, reaching approximately 2 kBq kg^–1^ as observed in this study, are attributed to the significant proportion of zirconium-based raw materials used, as it occurs, for example, in AZS (Alumina-Zirconia-Silica) blocks, which contains Al_2_O_3_-ZrO_2_-SiO_2_, used in glass furnace that contain typically 30–40% zircon (Wisniewski et al. 2019). In general, for the refractory products, values for ^238^U up to 2–3 kBq kg^–1^ were also reported in literature, whereas the typical concentration for ^232^Th series are generally lower than 1 kBq kg^–1^.

Regarding the **residues**, elevated concentrations of ^210^Pb (up to 37 kBq kg^−1^) and ^210^Po (up to 126 kBq kg^−1^) were found in samples of *fusion furnace dust* generated during the production of refractory and ceramics. In fact, the high process temperatures employed by the refractory industry (> 1900 °C) cause volatilisation of these nuclides.

For the tile industry, hydrated lime, a by-product of dust treatment from the firing process where the dust is extracted and precipitated using lime (Zampieri 2007), exhibited high ^210^Po concentrations (up to 46 kBq kg^−1^) and moderate concentrations of ^210^Pb (lower than 0.4 kBq kg^−1^).

Dust collected from the general air exhauster and scrap grinding operations showed ^238^U, ^226^Ra, and ^210^Pb concentrations exceeding 1 kBq kg^−1^. Additionally, scrap grinding dust contained ^210^Po at a concentration of about 1 kBq kg^−1^. These values agree with those found in the literature (IAEA 2007).

Wastewater sludge from brick and enamel production processes also contained considerable activity concentrations (approximately 1–2 kBq kg^−1^) of ^238^U series radionuclides.

Scrap materials (e.g., broken or defective objects, grinding residues, fired or unfired) from the refractory and ceramic industry exhibit activity concentrations comparable to finished products, showing elevated levels especially for ^238^U (up to 1.6 kBq kg^−1^) and ^226^Ra (up to 4.3 kBq kg^−1^).

### Cement production

Cement is a binding material widely used in construction to create concrete, one of the most versatile and durable materials, and an important backbone of infrastructure such as buildings, bridges, and roads (CEMBUREAU 2020). Concrete (and thus cement also, although alternative materials are developed and still worked on) is the second-most consumed substance globally after potable water, it is essential to socio-economic development (Czigler et al. 2020; IEA 2021). Cement is produced in a process that starts with the extraction of raw materials (e.g. limestone, clay) from quarries, continues with the production of clinker in specific kilns and concludes with the grinding of clinker with additives depending on the type of cement that is produced. Clinker production is the core of cement manufacturing, but it is also a very energy and intensive production phase in terms of (NO_*x*_, SO_2_, CO_2_) emissions. In manufacturing of cement, beside above-mentioned raw materials, some intermediate products from other industries can be used, such as coal fly ash, blast furnace slag, silica fume, steel, phosphorus slag, tin and copper slag and other (Puertas 2021). The main constituent in different cement types (e.g., Portland, blast furnace, pozzolanic and composite cement) is clinker, while residues commonly include the ash or dust generated during the production process due to kiln’s high temperatures. High production temperatures may give rise to NOR in the facilities equipment, such as dust filters, pipes, and kiln linings and especially release of ^210^Po (UNSCEAR 2000).

The overview of ranges and mean values of activity concentration of relevant radionuclides related to cement production is presented in the Table 4. Analysis of the scarce data available in this study, for raw materials, main product and residues showed no enhanced NOR activity concentration, i.e., it was comparable with published literature data (UNSCEAR 2000; Eštoková and Palaščáková 2013).

**Table 4:** Activity concentration of relevant radionuclides in different fractions of radiological concern related to cement production industry

Cement production industry								
Material type	^238^U	^226^Ra	^210^Pb	^210^Po	^232^Th	^228^Ra	^228^Th	^40^K
*1. Raw materials [kBq kg* ^*−1*^ *]*								
Pet coke	0.003	0.001	< MDA	< MDA	0.001	–	–	0.003
*2. Main products [kBq kg* ^*−1*^ *]*								
Clinker	0.03	0.025	< MDA	< MDA	0.016	–	–	0.26
*3. Residues [kBq kg* ^*−1*^ *]*								
Smoke abatement dust	0.002	0.02	< MDA	< MDA	0.01	–	–	0.2
Clinker scale in oven	0.01–0.39	0.026	< MDA–**1.4**	< MDA − **2.0**	< MDA–0.25	–	–	0.01–1.5
Scales from calcinatory	0.2–0.53	–	0.02–0.06	–	0.06–0.1	–	–	0.6–5.0
Spent Refractory brick	0.01–0.56	–	0.002–0.5	–	0.001–0.4	–	–	0.002–0.9

Activity concentrations higher than EU BSS ELs & CLs are highlighted in bold

Raw materials, main products and residues with activity concentrations higher than 1 kBq kg^–1^ for ^238^U and ^232^Th series, and 10 kBq kg^–1^ for ^40^K (for at least one radionuclide) are highlighted in bold

While the majority of mineral processing industries generate significant amounts of waste, the situation in cement production is quite the opposite. Certain residues are utilized as feedstock alongside natural raw materials, resulting in varying levels of NOR at different stages of the cement production process across different plants. However, a specific pattern of NORM activity concentration is observed.

Table 5 provides an inventory of NORM content in raw materials used for cement production and the final product, based on data collected from a cement plant over one production cycle. In the pre-processed raw material, the raw flour reflects the average NORM activity concentration in the feedstock according to the proportions used. In clinker, the activity concentration of radium and thorium isotopes is slightly higher due to loss on ignition (up to 50%). However, volatile ^210^Pb escapes from the process and ultimately accumulates in dust.

The NORM activity concentration in the final product (cement) depends on the additives used in the final mixture, which could include siliceous fly ash, granulated blast furnace slag, pozzolana, or limestone. Despite the accumulation process, the probability of the final NORM activity exceeding the established limits is rather low, unless extraordinary materials are added to the feedstock.

### Titanium dioxide (TiO_2_) pigment production

Titanium naturally occurs in chemical combination with oxygen and iron. Titanium dioxide (TiO_2_) is an oxide that is commonly found in Earth’s crust (Ayorinde and Sayes 2023). The key commercial titanium minerals include ilmenite (with TiO_2_ content of 34–69%); rutile (93–96.5% TiO_2_) and leucoxene with TiO_2_ content of 70–90%. More than 90% of titanium mineral production comes in the form of ilmenite.

Titanium minerals and various types of raw materials derived from them contain NOR from the decay series of ^232^Th and ^238^U. Radiological hazard associated to TiO_2_ production depend on the type and origin of the feedstocks. The principal producing countries for rutile and ilmenite are China, Australia, Mozambique, Sierra Leone, and South Africa (Geological Survey 2024). The overall manufacturing process consists in taking an impure TiO_2_ feedstock and converting it into pure white TiO_2_ pigment. To achieve this, the impure TiO_2_ must be chemically converted into another compound, impurities separated out and then converted back to pure TiO_2_ — in effect a chemical purification process (McNulty 2008; Martín Matarranz 2013; Gázquez et al. 2021).

The main uses of TiO_2_ pigment are in coatings, such as paints (around 60%), followed by plastics, high-grade papers, and printing inks. Thanks to its high refractive index, TiO_2_ is the best white pigment available. However, its use is not restricted solely to white colour applications; its opacity is also used in combination with coloured pigments to provide the required hiding power (McNulty 2008). Titanium minerals and the various process feedstocks derived from them contain radionuclides of natural origin form the ^232^Th and ^238^U decay series. The radionuclide activity concentrations are moderately elevated compared to those in normal rocks and soil. During processing, the radionuclides may become mobilized and migrate to dusts, scales and other process residues, potentially leading to radionuclide activity concentrations higher than those in the relevant feedstock mineral. Particularly, isotopes of radium may become concentrated in scales (IAEA 2012).

The process can follow either the ‘sulphate’ or the ‘chloride’ path and, in both cases, most of the radioactivity is located in the solid wastes of the process (scale deposits). There is no cause for concern regarding products, co-products, or liquid or gaseous effluents (McNulty 2008).

Data collated in this work included the waste materials such as red gypsum, iron sulphate, sludge, metal hydroxide, filter and digester residues, scales, acidic waste, ilmenite mud, brick lining, and dust emission. Iron sulphate can be reused as a fertilizer (Table 5). It is an iron-rich, fast-acting, and long-lasting fertilizer that stimulates strong grass growth and gives it a healthy, dark green colour.

**Table 5:** Inventory of raw materials used for cement production based on an example of one technological process

Cement production based on an example of one technological process					
Material type	^226^Ra	^210^Pb	^228^Ra	^228^Th	^40^K
*1. Raw materials[kBq kg*^*−1*^]					
Base stone (limestone)	0.025–0.028	0.012–0.02	< 0.002	0.001	0.014–0.02
Iron-bearing additive	0.008–0.009	0.015–0.022	0.003–0.005	0.003–0.004	0.028–0.036
Ground blast furnace slag	0.088–0.099	< 0.027	0.024–0.029	0.028–0.032	0.1–0.12
Coal ash	0.12–0.14	0.13–0.15	0.079–0.096	0.078–0.094	0.68–0.79
Coal	0.029–0.035	0.036–0.044	0.021–0.026	0.021–0.026	0.157–0.187
Alternative fuel	< 0.002	< 0.008	< 0.006	0.002	0.007–0.026
Gypsum stone	0.015–0.017	0.011–0.014	0.004–0.005	0.004–0.005	0.059–0.07
Chromium reducer	0.021–0.025	< 0.025	0.021–0.024	0.024–0.028	0.38–0.45
Raw flour	0.017–0.019	0.001–0.019	0.009–0.012	0.01–0.011	0.161–0.193
Clinker	0.035–0.039	0.003 −0.015	0.021–0.024	0.019–0.022	0.213–0.251
*2. Main products [kBq kg* ^*−1*^ *]*					
CEMII/A-V 42.5R cement	0.039–0.044	0.02–0.04	0.025–0.029	0.025–0.036	0.239–0.283
*3. Residues [kBq kg*^*−1*^]					
Portland cement production dust*	0.02–0.024	0.484–0.534	0.011–0.015	0.011–0.013	1.74–1.96

*This material is a residue of the cement production process and is subsequently reintroduced into the manufacturing process

Activity concentrations of NOR in the **raw material** presented in Table 5 are in a good agreement with those presented by McNulty (2008), where rutile with a high grade of titanium dioxide (93–96%) contains 0.1–0.74 kBq kg^−1^ of ^238^U and 0.08–0.36 kBq kg^−1^ of ^232^Th; ilmenite with TiO_2_ (45–65%) contains 0.1–1.0 kBq kg^−1^ of ^238^U. Activity concentrations higher than 1 kBq kg^−1^ for at least one radionuclide have been found for ilmenite sand and rutile. As for the **main product** as TiO_2_, the activity concentration of NOR are usually insignificant, which coincides with other sources (IAEA 2012; Mrdakovic Popic et al. 2023b). Regarding the **residues** activity concentrations higher than 1 kBq kg^−1^ for at least one radionuclide have been found for scale, sludge, rubberisation, brick lining of tanks, ilmenite mud, surface material of tank lining, filter residue, metal hydroxide, wet mud. Significant activity concentration of NOR can be found in scales, where the upper-level values of NOR activity concentration are in a good accordance with the e-NORM survey (Mrdakovic Popic et al. 2023b), although in the latter source, the concentration of ^226^Ra and ^210^Pb are higher and the concentration of ^232^Th lower than those presented in Table 5. In relation to sludge, the NOR of highest importance are ^226^Ra, ^232^Th, ^228^Ra. As expected, such residues as filter material could have increased activity concentration of NOR, especially in radionuclides like ^210^Pb, ^210^Po, ^232^Th and ^226^Ra.

In addition, high radon concentrations were reported in some works (Gazquez et al. 2021) measured in crystallizers (about 1000 Bq/m^3^), what need a forced ventilation during the maintenance operations to reduce the radon concentration to values lower than reference level 300 Bq/m^3^ for working places recommended by EU BSS.

**Table 6:** Activity concentration of relevant radionuclides in different fractions of radiological concern related to TiO_2_ production

TiO_2_ production								
Material type	^238^U	^226^Ra	^210^Pb	^210^Po	^232^Th	^228^Ra	^228^Th	^40^K
*1. Raw materials [kBq kg*^*−1*^]								
Ilmenite sand	0.05–**2.0**	0.005–**3.7**	0.001– 0.1	–	0.1–0.4	0.2–**1.65**	<0.005–**1.6**	0.002– 0.1
Ilmenite rock	0.1–0.13	0.063–0.1	–	–	–	0.2–0.4	0.2–0.4	–
Unused brick for lining	0.061	0.051	0.04	–	–	0.064	0.062	0.6
Quartz sand	<0.05	<0.005	–	–	–	<0.005	–	<0.01
Rutile	0.06–**1.1**	–	–	–	0.03–**1.8**	–	–	–
Titanium slag	0.09	0.006–0.03	0.01–0.08	–	0.02–0.05	–	–	0.01–0.2
Zirconium beads	**3.6**	**2.7**	–	–	–	0.78	–	<0.01
*2. Main products [kBq kg*^*−1*^]								
Titanium dioxide	0.0008–0.2	0.001–0.05	–	–	–	0.002–0.02	0.001–0.007	<0.002–0.04
*3. Residues [kBq kg*^*−1*^]								
Scale	0.015–**1.6**	0.05–**25.0**	**37.4**	–	**493.7**	0.075–**37.0**	–	0.008–2.6
Sludge	0.55	**4.3**	0.55	–	**14.5**	**2.3**–**2.9**	–	1.4
Piping	0.03	0.04	0.03	–	–	0.047	0.06	0.06
Rubberisation	<0.2–0.6	0.25–**4.1**	0.65–**2.3**	–	–	0.09–**5.2**	0.15–**6.6**	0.2–0.24
Brick lining of tanks	0.06–0.75	0.07–**6.2**	0.06–**2.3**	–	–	0.1–**4.8**	0.12–**5.9**	0.6–0.8
Iron sulphate	0.04– 0.06	0.007–0.01	–	–	0.3–0.4	0.03–0.05	–	–
Ilmenite mud	0.16–**4.3**	0.007–**21.0**	–	–	–	0.01–**8.3**	0.06–**1.4**	0.017–0.084
Dust filter	<0.005	<0.001	–	–	**150.0**	<0.001	–	<0.001
Surface material of tank lining	**4.5**	**48.0**	–	–	–	–	–	–
Filter residue	**7.0**	0.05–**35.6**	**3.2**–**187.0**	**133.0**	**87.7**	0.05–**20.5**	–	0.12–1.9
Filter press residue	–	0.3– 0.5	–	–	0.3–0.4	–	–	–
Metal hydroxide	**2.0**–**8.0**	–	–	–	**2.0**–**8.0**	–	–	–
Red gypsum	0.016– 0.02	0.009–0.02	–	–	0.1–0.2	0.08–0.1	0.09–0.16	–
Wet red gypsum	0.02	0.01	0.08	–	0.15	0.09	–	0.01
Wet mud	0.2	0.82		–	0.353	**2.6**	–	0.28
*4. Liquid [kBq kg*^*−1*^*] and gaseous effluents/[Bq m*^*−3*^]								
Liquid acidic effluent	0.16	0.1	–	–	–	0.014	0.2	–
Atmospheric release	0.006–0.086	0.002 −0.009	–	–	0.21	0.002	0.001	–

Activity concentrations higher than EU BSS ELs & CLsare highlighted in bold

Raw materials, main products and residues with activity concentrations higher than 1 kBq kg^–1^ for ^238^U and ^232^Th series , and 10 kBq kg^–1^ for ^40^K (for at least one radionuclide) are highlighted in bold

## Discussion and challenges

To identify critical materials where radiological aspects may be relevant and for the purpose of comparison between different mineral processing industries, an overview of raw materials, products and residues containing NORM with elevated levels of some isotopes from ^238^U, ^232^Th decay chains, and ^40^K is presented in Table 7.

**Table 7:** Overview of NORM materials by industrial sector with samples exceeding ELs

Industrial sector	Raw materials	Products	Residues
Phosphoric acid	Sedimentary rock (Morocco) Potassium salt	Phosphoric acid 54%	PG Scales Liquid and solid sludge Filtres
Phosphate and potassium fertilizers industry	Sedimentary rock (Tunisia, Morocco) Phosphate ore Potash ore Apatite Phosphoric acid K_2_SO_4_ Muriate of potash	Monoammonium phosphate Diammonium phosphate	Scale Calcium fluoride sludge
Zirconium industry	Zircon sand	Refractory	Fusion furnace dust
	Zirconium oxide	Refractory slurry	Hydrated lime
	Silica fume	Zirconium beads	General air exhauster dust
			Scrap grinding dust
			Sludge
			Scrap refractory residues
TiO_2_ industries	Ilmenite sand Rutile		Scale Sludge Rubberisation Brick lining of tanks Ilmenite mud Surface material of tank lining Filter residue Metal hydroxide Wet mud
Cement production industry			Clinker scale in oven

Cement industry is not included in this table as no NORM with radionuclide activity concentrations > EU BSS numerical Els and CLs were reported in the current study

In all the industrial sectors surveyed, it was found that there are some residues with NOR activity concentration above the exemption levels of the EU BSS and of a certain radiological significance. The destination of these residues involves in some cases their disposal in a dedicated landfill, conventional landfill or landfill for hazardous waste, such as for phosphogypsum, hydrated lime, scales or sludges. The reuse of other residues within the same plant, such as clinker scales or abatement dust from refractory production, has also been observed. Furthermore, other residues are subject to treatment outside of the plant where they have been created. Besides disposal, many efforts are spent in the recovery of materials following circular economy rules, especially with PG. These aspects will be investigated in more details in next work.

A common aspect to the management of these residues is, to some extent, that the treatment operations are not carried out by workers of the company producing them. This is exemplified by the case of landfill workers who are managed by specific companies. Another significant example concerns clinker scale removal operations, which are typically executed by workers from specialised servicing companies external to the cement factory.

The radiation protection of these workers is not generally considered by the companies producing the waste, nor by the employers themselves, since they are not industrial sectors strictly included in the list of Annex VI of the EU BSS. However, the work activities as previously described can be traced back to the relevant secondary processes, to which Annex VI itself refers and to which the same requirements apply as to NORM involving industrial sectors. This includes, therefore, the graded regulatory control approach to ensure radiation protection. This issue has already been highlighted by Michalik et al. (2023) and certainly needs to be further investigated, in order not to overlook relevant exposure pathways for workers and members of the public.

The study also underscores the necessity of direct attention towards end products as previously emphasised (Michalik et al. 2023). As illustrated by the data presented in the tables of Sect. 4, there are notable activity concentrations in certain end products, such as phosphate fertilizers and refractory materials. It is noteworthy that the EU BSS does not set threshold limits for marketing of these products. Instead, the EU BSS calls upon Member States to implement measures aimed to protect the public and workers (Art. 100 EU BSS, Annex XVII). The data collected can facilitate the development of screening tools based on product life cycles, encompassing their wholesale trading, transport, storage, application and use, final disposal and also secondary wastes generated.

To illustrate this point, refractory bricks for glassworks furnaces can be considered. Within the life cycle of these materials, production at the company should be taken into account, as well as all activities related to their use and disposal. Such activities include the construction of the furnaces, the demolition and the treatment of the demolished refractory bricks. The definition of screening levels should, therefore, encompass all these aspects and the corresponding exposure scenarios in order to ensure the radiological safety of products throughout their entire life cycle. In the context of mineral processing industries, particular attention should be directed towards phosphate fertilizers and refractory materials. Titanium dioxide and phosphoric acid generally do not have relevant activity concentrations, while cement and ceramics are already regulated as building materials in the EU BSS.

The illustrative examples of exposure scenarios of radiation protection concern from certain mineral industries are presented in Table 8, together with applied countermeasures.

**Table 8:** Critical points for certain industries associated with radiation protection

Industry	Critical point/situation	Countermeasure
Phosphoric acid	The accumulation of large amounts of phosphogypsum can cause noticeable or significant environmental risks	Control of accumulation of phosphogypsum and management it in the proper way
Titanium dioxide industries (TiO_2_)	High radon activity concentrations measured in crystallizers (about 1000 Bq/m^3^)	Forced ventilation during the maintenance operations is needed to reduce the radon activity concentration to values lower than 300 Bq/m^3^ for working places
Zirconium industry	Radiological exposition of workers involved in the building/demolition of refractory brick furnace; radiological exposition of workers involved in the management of wastes from demolition of refractory bricks	Definition of criteria for relevant secondary processes in order to extend radiation protection to workers not closely involved in the production process
Cement production	Radiological exposition of workers involved in the maintenance of the kiln (clinker scales removing and kiln lining repairing)	Definition of criteria for relevant secondary processes in order to extend radiation protection to workers not closely involved in the production process

In addition, it should be mentioned that in this study the activity concentration of NOR were collected for mineral processing industries as NORM inventory for further consideration in Life Cycle Assessment. In European Commission 2011; Annex 2 research needs to take into account impact of ionizing radiation category in LCA are identified and prioritized. One of the relevant tasks for such development of ionising radiation category is mentioned as “Extend the number of radionuclides covered for both human health and ecosystems”, which is also considered as “high workload” (European Commission 2011; Annex 2: Research needs). To some extent, the present work addressed this requirement, in addition to other aspects, as the data for different NOR such as ^238^U, ^226^Ra, ^232^Th, ^40^K, ^210^Pb, ^228^Th, and ^228^Ra have been collected and analysed.

**Fig. 2:**
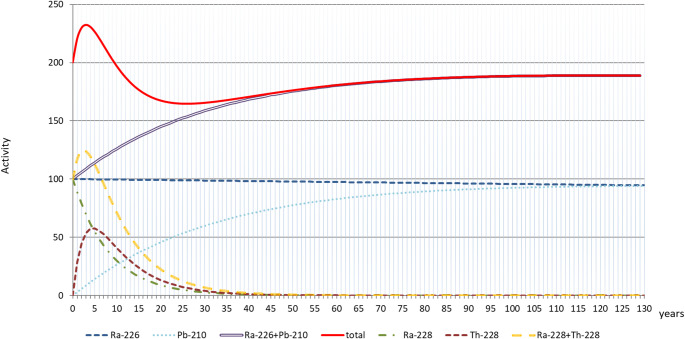
Relative changes (in %) of NOR composition and activity concentration in material where at the beginning only radium isotopes ^226^Ra and ^228^Ra were present, e.g. scales precipitated from radium rich water

Collected data set is providing an additional opportunity to discuss the overall quality of data concerning NOR activity concentration. Quantitative data presented in tables were provided by organisations participating in RadoNorm project and taken from scientific article published in reviewed journals. As there is usually no supplementary data concerning the history of material sampled (i.e. when it was created, deposited etc. in general, enabling to recognise how old a sampled material was), they are considered as reflecting the current state of sampled material. Given that all NOR considered (besides ^40^K) are members of a decay series and they are subject to successive decay, it is necessary to assume factual NOR activity concentration is changing according to the passage of time. Moreover, the composition of NORM may change, depending on the time horizon assumed, as a new decay product will appear which were not present at the moment of material of concern creation (Fig. 2). It implies that for the proper evaluation of residue properties a forecast is necessary, especially for processed materials where a probability exists that secular equilibrium in the decay series has been disturbed (Michalik et al. 2013). As processes of residues recovery and, especially disposal must be considered in specific time horizons such forecast can influence the final evaluation/decision significantly.

The rules of successive decay also let one to complete data concerning radionuclides composition when not all NOR, that may contribute to the overall exposure scenario, have been directly measured. In general, for the unprocessed, solid, raw materials the state of radioactive secular equilibrium can be assumed, even though only one radionuclide, even having very short half-life (e.g. ^214^Bi in uranium decay series) is measured. Hence, for the further evaluation long-lived members of the decay series must be considered. In case of processed materials (residues) this assumption is not valid at all and for relevantly long-lived radionuclides measured it is necessary to consider all decay products, however there is no possibility to conclude about a parent radionuclide. E.g. measuring ^228^Ra in a sample one has to consider also ^228^Th in a few years perspective, however there is not enough premises to conclude about ^232^Th activity concentration. The assumption about equilibrium state let one also make a results quality cross check (Karfopoulos et al. 2025). Apart from decay law, the properties of isotopes of an element determine a material composition e.g. there is no option to get processed material containing ^232^Th with significantly lower content of ^228^Th. The lowest possible ^228^Th/^232^Th activity ratio is about 0.4, unless deliberate isotopes separation is applied.

## Conclusions

Naturally occurring radioactive materials (NORM) in mineral processing industries such as phosphoric acid production, phosphate and potassium fertilizers, zirconium, cement production, and TiO_2_ industry were investigated. The ranges of the activity concentrations from raw materials, different production processes, end products, residues and wastes containing NORM, as well as an overview of the NORM and exposure scenarios of radiation protection concern were collected and compared.

The main conclusions can be summarised as follows:

- Legislative requirements from the EU BSS are applied in the here investigated NORM involving mineral industries, with certain degree of differences in graded regulatory control resulting from the flexibility allowed in the EU BSS in order to account for specific national conditions.
- In all industries investigated, with the exception of the cement industry, NOR activity concentration of raw materials, such as sedimentary rocks, phosphate and potash sands, zircon sand and zirconium oxide, are exceeding the ELs in many cases, although the values are in a wide range therefore greatly depending on the origin of used raw materials.
- Main products in all investigated industries are mainly of no radiation protection concern as reported NOR activity concentration were below 1 kBq kg^−1^ and 10 kBq kg^−1^ for ^238^U and ^232^Th decay chains and ^40^K, respectively. However, it was established that a degree of attention should be allocated to certain products, including fertilizers and refractory materials, which have been observed to contain non-negligible activity concentration of NOR. In this regard, the information collated here may be of use in the development of screening tools, with a view to providing a basis for the assessment of the radiological impact on the population.
- Regarding residues, phosphogypsum is the most abundant NORM, however NOR activity concentration usually are not high, although sometimes can exceed the ELs. On the other hand, significantly lower amounts of scales and sludges are present in these industries, but with some high reported radionuclide values. The main radionuclide of concern in these cases are ^226^Ra and ^210^Pb, ^228^Ra and ^228^Th.
- The data collected for the cement industry is limited and by no means exhaustive. Indeed, this is the latest NORM industry to be introduced by the EU BSS, and information regarding radiological aspects remains limited.
- The data collected on liquid and gaseous effluents is also very limited for all industries. Additionally, the information on such effluents is expressed in different units, sometimes in activity concentration of NOR in effluents (kBq/kg or Bq/m^3^) or as a batch (total activity) discharged per year (Bq/year), making comparison and data management more difficult. Therefore, further research on this issue is needed in future works.
- The analysis revealed the presence of certain recurring themes within all the sectors of the mineral industry. It was emphasised that the analysis of radioprotection aspects should be expanded beyond the confines of production facilities, encompassing the exposure of workers involved in the recovery, recycling, or final disposal of residues, as well as those engaged in maintenance activities of plant components (e.g., clinker kiln scale removal).
- The quantitative information presented in this work can be utilized to complement, compare and improve existing data in LCI databases as e.g. using the NOR activity concentrations in the materials and products related to the above mentioned mineral processing industries at different stages of production. Furthermore, qualitative information, such as the types of materials and wastes that contain NOR, their destination, possible use or reuse can be utilized to make the LCI databases more complete and create more realistic LCA scenarios. Finally, such information can be used to estimate the impact of ionising radiation on human health in terms of radiation doses in different phases of the lifecycle of the product or services (more information of this will be presented in the Part II).

Regardless the main topic discussed it must be underlined that the urgent needs are identified to develop a standardised procedure for NORM characterisation, including reference measurements techniques, scope of measurement and measurement results interpretation. This article presents a first part of a comprehensive work on NORM in mineral processing industries, and is focused on NORM inventory. In a coming work, the second part of the paper, an exhaustive analysis of NORM ionising radiation impact in these industries and approach to consider such impact in Life Cycle Assessment will be presented.

## Data Availability

No datasets were generated or analysed during the current study.
